# “Know your audience”: A hospital community engagement programme in a non-profit paediatric hospital in Cambodia

**DOI:** 10.1371/journal.pone.0182573

**Published:** 2017-08-03

**Authors:** Sreymom Pol, Shivani Fox-Lewis, Phaik Yeong Cheah, Claudia Turner

**Affiliations:** 1 Cambodia Oxford Medical Research Unit, Siem Reap, Cambodia; 2 Mahidol Oxford Tropical Medicine Research Unit, Faculty of Tropical Medicine, Mahidol University, Bangkok, Thailand; 3 Centre for Tropical Medicine & Global Health, Nuffield Department of Medicine, University of Oxford, Oxford, United Kingdom; 4 The Ethox Centre, Nuffield Department of Population Health, University of Oxford, United Kingdom; 5 Angkor Hospital for Children, Siem Reap, Cambodia; Waseda University, JAPAN

## Abstract

**Objective:**

The purpose of this evaluation is to explore the impact of the new hospital community engagement programme (comprised of a Young Persons Advisory Group and a Science Café) on community members and other stakeholders, with regard to their attitudes, skills and degree of engagement in a paediatric hospital in Cambodia.

**Design:**

Data collection included feedback questionnaires and reflections produced after each YPAG and Science Café event. Further questionnaires and reflective interviews were conducted to gather the views of key stakeholders. Data were analysed by thematic content analysis and numerical data were expressed using descriptive statistics.

**Results:**

The vast majority of participants expressed their enjoyment and satisfaction of the hospital community engagement programme. Delivering the programme in the right manner for the target audiences, by prioritising their needs was key to this. Participants valued the programmes in terms of the knowledge delivered around good health practices, the skills developed such as confidence and responsibility for their health, and the provision of opportunities to voice their opinions. All stakeholders recognised the importance of the programme in improving the quality of the healthcare service provided at the hospital.

**Conclusions:**

In order to have a successful hospital community engagement programme, understanding the target audience is essential. The engagement programme must be delivered in the right way to meet the needs of community members, including right communication, right setting, right people and right timing. This will ultimately result in a meaningful programme that is able to empower community members, potentially resulting in lasting change in healthcare practices. In conclusion, the gap between hospitals and the community could narrow, allowing everyone to interact and learn from each other.

## Introduction

Cambodia was classed as a “lower-middle income” country in 2015 by the World Bank, however the majority of the population are still living in poverty or near poverty [1]. The vast majority of the population live in rural areas [2]. The median under-five mortality rate in 2015 was 28.7 per 1000 live births [3]. The structure of healthcare provision relies on health centres in rural communities, with fewer referral hospitals (at a provincial and district level). Running alongside these are private and non-governmental facilities, and traditional healthcare practices also play a role [4].

Since 1999 Angkor Hospital for Children (AHC) [4], located in Siem Reap in north-western Cambodia, has been providing free healthcare to children < 16 years old that present to the hospital. During that time the hospital has expanded to include outpatient, inpatient medical and surgical, neonatal and intensive care facilities. The hospital’s aim has always been to provide quality compassionate care, and with this in mind a hospital-based community engagement programme was launched in 2015.

The hospital community engagement programme at AHC was developed to meet the specific needs of the community served, in order to improve the quality of the care provided at the hospital. Two ‘sub-programmes’ were developed to target adults (Science Café) and children (Young Persons Advisory Group), both organised and facilitated by the author SP.

The Science Café (SC) was designed to primarily engage adults in matters important to their own health and that of their children. Most participants are illiterate agrarian workers with little to no formal education. The format of the SC model is such that events are usually held in casual settings and science is discussed at friendly and traditionally non-academic venues like cafés, bars, and theatres [5–7]. Speakers are asked to produce a PowerPoint presentation with only pictures on their slides and to explain concepts using lay language, speaking in Khmer (the national language of Cambodia). Following the talk (usually 20 to 30 minutes) there is an opportunity for audience members to discuss amongst themselves and ask the speaker questions. This discussion occurs in an informal manner, with food and drinks provided during this time. The speakers are healthcare staff from AHC and the audience members are caregivers of patients, and sometimes the patients themselves. The SC is advertised using posters displayed in and around the hospital, and on the day of the event the organiser informs parents present in the hospital’s waiting areas and wards in case they wish to attend. The aim of the SC is to increase awareness of health-related topics, and to help the community members feel more comfortable in discussing such topics in their own way. In addition the SC helps to bridge the gap between healthcare staff and their audience by providing a relaxed environment in which community members can interact with hospital professionals.

The Young Persons Advisory Group (YPAG) was designed to engage children aged 10 to 15 years old [8] in matters important to their community and to AHC. Whilst YPAG initiatives have been developed in other settings [9–12], there is a paucity of published literature on their efficacy. The YPAG was advertised throughout the hospital and children could volunteer to join. Participants are mainly the children of staff members and their friends (via a ‘snowball’ recruitment effect). The young people that are part of the YPAG are able to reflect the views of a key part of the community of particular importance to AHC as it is a paediatric hospital. Enabling these key stakeholders to take an active role in the organisation will allow AHC to continue to provide quality care to its patients. The participants are encouraged to work together to determine which activities they want to conduct, and what their priorities are for improving the care provided by the hospital. The group provides a platform to incorporate the views of young people into how the hospital is run, how it could be better suited to their needs and the research activities occurring at AHC. The YPAG aims to empower young people to express their views to their peers and to those in positions of authority.

The purpose of this formative evaluation is to determine the perceptions of the hospital community engagement programme from participants and other key stakeholders. This evaluation explored the impact of the YPAG and the SC on community members, with regard to their attitudes, skills and degree of engagement in hospital-related topics.

## Methods

The hospital community consists of children and adults, therefore the hospital community engagement programme was designed to address both target audiences (via YPAG and SC respectively). Since both YPAG and SC have common aims and common features to their implementation, they will be presented together, as facets of one hospital community engagement programme.

### Setting

The hospital community engagement programme is conducted at Angkor Hospital for Children, Siem Reap, Cambodia [4]. Key stakeholders for this evaluation included the SC audience, the YPAG members, the organiser of SC and YPAG (author SP was organiser for both), the speakers at SC, the parents of YPAG members and the AHC Executive Committee. The SC occurs every two months, with 30 to 40 audience members in attendance each time. The first SC was held in August 2016. The YPAG consists of 20 members and meets every month, commencing February 2016, with additional ad hoc events as required.

### Study design

This mixed methods evaluation was conducted from February to April 2017. Data collection included feedback questionnaires and reflections produced after each SC and YPAG event. Additional data were collected specifically for this evaluation to capture the views of all key stakeholders. Table 1 shows the various data sources and methods of data collection.

**Table 1:** The data collected from different stakeholders.

Sub-programme	Data source	Subject of data
**SC & YPAG**	Reflection	Completed by SC and YPAG organiser
**SC & YPAG**	Questionnaire*	AHC Executive Committee
**SC & YPAG**	Events log	YPAG meetings and events
**SC**	Reflection	Speakers at SC
**SC**	Questionnaire	Audience members
**YPAG**	Reflective interview & Questionnaire*	YPAG members
**YPAG**	Questionnaire*	Parents of YPAG members

*Data collected purposively for this evaluation

### Data collection and analysis

The organiser wrote reflections after each YPAG and SC event. For SC these reflections included the date, location, topic and number of audience members, followed by the organiser’s views of the event, including the level of audience engagement. After the event the organiser followed-up with the speaker to obtain their reflections on the event verbally. This speaker’s reflection was typed and included in the organiser’s reflection document.

At each SC event, audience members were asked for their feedback using a structured questionnaire, and the evaluation team recorded their answers by taking notes. Questions included their knowledge of the topic before and after the talk, whether they understood the talk, whether they enjoyed the event, what they found good and what they would improve about the SC. These questionnaires were completed by Khmer-speaking members of the research team at the Cambodia Oxford Medical Research Unit (COMRU), who randomly sampled 20% of audience members. Reflections on YPAG events were written after the event by the organiser and included the number of YPAG members present, a description of the activities that occurred and the organiser’s impressions of the event.

The events log for SC and YPAG was kept by the organiser as a record of when events were held, what they were and how many participants were involved.

To ensure that the views of all key stakeholders were incorporated in this evaluation, data were purposively collected from the AHC Executive Committee. The Executive Committee comprises the Chief Executive Officer, the Hospital Director, the Chief Operating Officer and the Chief Business Officer. Questionnaires in English were given to each of the four members, with questions including whether Executive Committee members were aware of the YPAG and SC, their opinions of them, what kind of outputs they would like to see from these programmes and how they think the programmes could be improved.

To capture their views, all 20 YPAG members were asked to complete a questionnaire translated into Khmer, which included questions on why they chose to join the YPAG, what they feel they have got out of it, the impact it has had on them, and their perception of the impact YPAG has had on the hospital. Specifically, YPAG members were asked whether they felt more confident, able to work in a team, more responsible and whether they felt their voice was being heard as a result of YPAG. Furthermore, reflective interviews were conducted with some of the YPAG members, to gain greater insight into the impact of YPAG. The reflective interviews consisted of a relaxed conversation in Khmer between the organiser and individual YPAG participants, one at a time, lasting approximately 15 minutes each. The conversation covered the topics of what aspects of YPAG they enjoy, what can be improved and what changes participants have seen in themselves as a result of YPAG. The organiser recorded participants’ answers by taking notes.

Similar questions were posed to the parents of the YPAG members via a questionnaire, also translated into Khmer, to ascertain their perceptions of the impact of the YPAG on their children.

Numerical data were entered into a Microsoft Excel 2013 spreadsheet and simple statistics applied to describe the number of events, number of participants and age and gender of participants. Data from the reflective interviews were typed into a Microsoft Word document. Data from questionnaires were entered into a Microsoft Excel 2013 spreadsheet. The Microsoft Word and Excel documents were imported into a qualitative software package (NVivo qualitative data analysis software; QSR International Pty Ltd. Version 11, 2015). Thematic analysis was conducted, using a data-driven inductive approach. The coding framework was revised and refined by the study team, as key themes within the data were realised. Data were collected in an anonymised manner, no names of respondents were recorded. All data in a paper format were stored in a locked filing cabinet at the secure COMRU office. All digital data were password-protected and stored on the secure COMRU server.

### Ethical considerations

As this project is a programme evaluation both the AHC Institutional Review Board (AHC-IRB) and the Oxford University Tropical Research Ethics Committee (OXTREC) have declined the need for review and approval. The evaluation was conducted as per the protocol, in accordance with the principles of the Declaration of Helsinki and Good Clinical Practice, and adhered to the Research Governance policies of the University of Oxford and any other applicable requirements. The Memorandum of Understanding between AHC and the University of Oxford includes agreement for scientific collaboration between AHC and COMRU.

## Results

Overall, 31 audience members from the SC participated in questionnaires, four SC presenters gave their feedback, 20 YPAG members participated in questionnaires and 10 in reflective interviews and 10 of the YPAG parents and four Executive Committee members completed questionnaires. The key themes emerging from this data are presented below.

### Overview of the hospital community engagement programme

Since the SC began in August 2016 there have been four meetings covering the following topics: cancer in children, pneumonia in children, antibiotic use, and the science behind medication. On average there were 35 community members present at each meeting (range 30 to 45). In total 31 community members provided feedback on the SC via the questionnaires; their views are presented in Table 2.

**Table 2:** Community members’ feedback after each Science Café event.

SC General information	
Sex Female n (%)	22 (71)
Average age (min-max) years	41 (20–68)
The event is n (%)	
Fun	30 (97)
Neutral	1 (3)
Not Fun	0 (0)
How often would you want a SC meeting? n (%)	
Every two months	3 (9.5)
Every month	25 (81)
Every two weeks	2 (6.5)
Every week	1 (3)
How easy to understand was the topic? n (%)	
Easy to understand	25 (80)
I understood 50%	5 (17)
I understood less than 50%	1 (3)

From the survey results, 97% of the audience considered the Science Café session as fun, and the majority found the topics easy to understand. Most community members requested to have more frequent SC meetings, with the majority wanting monthly meetings.

Since the YPAG began in February 2016 there have been 12 monthly meetings and four events. During these regular meetings YPAG members conducted team activities and also planned their participation in additional events, such as football with the hospital, the Angkor Wat international half marathon, and their participation in international children’s day. Over one year the number of YPAG members has increased from 10 to 20. On average 16 members attended each monthly meeting. Of the 20 current YPAG members, all completed a questionnaire and 10 participated in reflective interviews.

**Fig 1:**
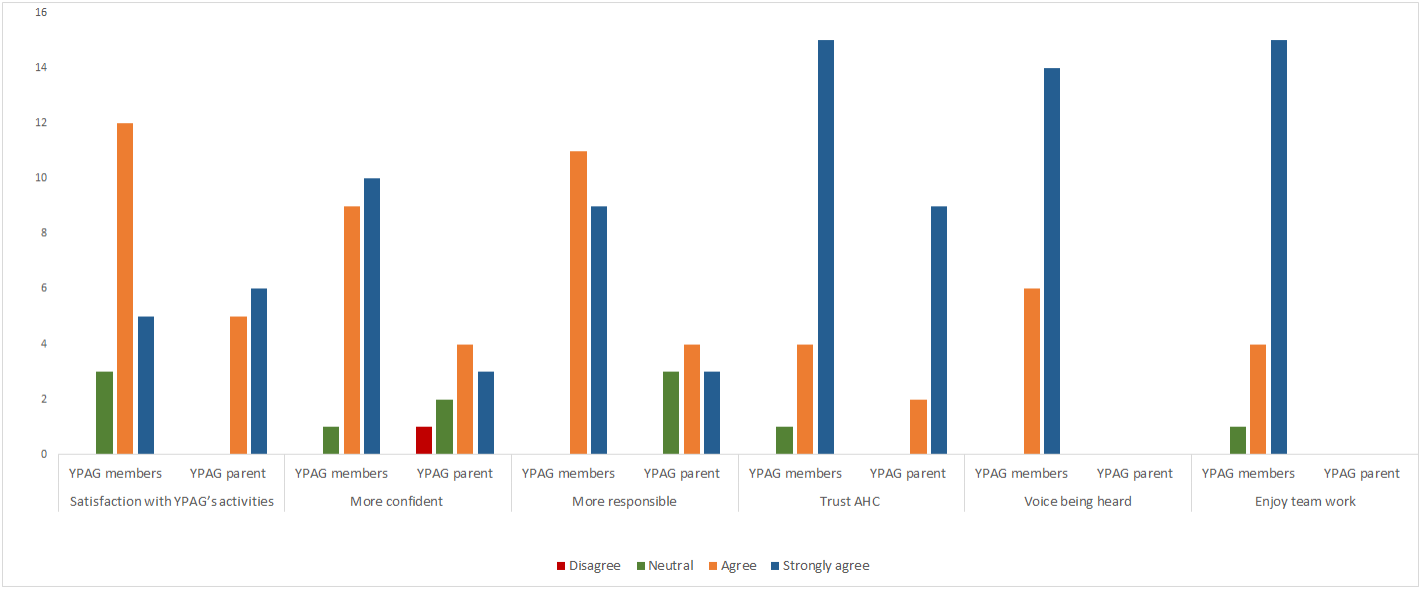
Opinions of the YPAG member and their parents.

Fig 1 shows the views of the YPAG members and their parents regarding the impact that participation in YPAG has had on the YPAG members (Fig 1).

Fig 1 clearly shows that YPAG members and their parents feel positively towards the programme, in terms of their satisfaction with it (Fig 1). The vast majority of YPAG members feel they enjoy team work and that they are more confident and responsible since being part of YPAG. Importantly, all YPAG members feel that their voices have been heard. Their parents also agreed that YPAG had a positive impact on their children in terms of developing confidence and responsibility. All respondents indicated that they trust AHC, which is essential for the programme to function (discussed below in “Trust”).

The evaluation also shows that the YPAG programme has had a positive impact on developing the specific attributes of confidence, responsibility and team-working within its members. One of the aims of the programme was to empower its members to realise and voice their views, and developing these attributes is a key part of this.

Qualitative analysis of the data presented eight key themes: improving the quality of care, passion and determination, attitude and empowerment, trust, communication, teamwork, suggestions for improvement and unintended consequences of the engagement programme. These will be discussed below.

### Improving the quality of care

The hospital community engagement programme aims to improve the quality of the healthcare services provided by AHC. Over one year, the Young Persons Advisory Group (YPAG) group conducted two inspections of the hospital and patient satisfaction surveys. They designed questionnaires and conducted a cross-sectional satisfaction survey with the patients aged 7 to 15 years who were admitted to the hospital. They then presented the results of the surveys to the senior management team of the hospital, which included Executive Committee members. In doing so, YPAG members were able to work as a team, assimilate patients’ views and present them to those in positions of authority. The information provided helped AHC improve the care provided, and the experience of doing this was a key part of developing essential attributes within the YPAG members, to further enable them. For example, one of the outcomes from the patient satisfaction survey was that patients would value a playground and a library, projects which are under consideration by senior management teams at AHC.

Participants said that the coming to the SC increased their knowledge and resulted in change in health practices for them and their children, for example by using medication correctly:

I know more about how to use medicines correctly, and I would not now buy any medicine without consulting an expert.

(Science Café, Audience)

Improving caregivers’ understanding of disease processes and medical care will greatly improve the overall care provided by AHC, as caregivers and healthcare staff will have a shared understanding of how to care for the patients.

The presenters themselves also said that SC helped them because they had the opportunity to talk to the caregivers:

I liked this session because I got the chance to talk to parents in more detail than when I see them in my clinic. In the clinic there is often not enough time to explain because there are many other patients waiting to be seen.

(Science Café, Presenter)

The hospital community engagement programme is able to improve the quality of care provided at AHC by engaging two essential groups: children and caregivers. However, it is the passion and determination of the key stakeholders that drives the programme.

### Passion and determination

A key reason for the success of the community engagement programme in involving participants in improving the care provided by AHC is that the participants themselves demonstrated great passion and determination to do so. When asked why they wanted to be part of YPAG, the young people commonly said it was because they wanted to help make the hospital better.

Because I want to play a part in making the hospital better, to help the patients.

(YPAG member)

Enthusiasm and commitment from key stakeholders is essential for success of the community engagement programme. For example, this SC presenter recognised that the opportunity to speak at SC improved their presentation skills:

I liked the experience because I could practice my communication skills.

(Science Café, Presenter)

Also crucial is the recognition by participants that the programme is beneficial to them. One YPAG member expressed how important YPAG is to them:

I really want the Cambodian people to really see the potential in children.

(YPAG member)

The above quote highlights the importance of participants recognising the value in their participation and what they have to offer. The hospital community engagement programme is able to empower community members because they themselves see that it has the potential to do so, and are committed to achieving this.

### Attitudes and empowerment

Participants’ responses in this evaluation demonstrated that the hospital community engagement programme has educated and empowered community members to actively participate in their healthcare.

Science Café has increased communication between healthcare professionals at AHC and community members by creating a relaxed environment in which they can ask questions. For example this presenter reflected on how engaged the audience were with the topic presented, asking appropriate and interesting questions, such as in the quote below.

During lactation, if the child cannot take the medicine, can the mother take the medicine instead and breastfeed the child?

(Science Café, Presenter)

The culture in Cambodian society is commonly not to question those seen to be in positions of authority, and as such caregivers rarely ask questions of healthcare staff in practice. Therefore providing an environment in which they feel comfortable to do so is a key step in improving their knowledge and understanding. As these audience members explain:

I really like this event because I feel that it overcomes the usual power imbalance between us and the hospital professionals, such that here we can interact more freely together.

I feel comfortable asking questions back and forth with the presenters.

(Science café, Audience)

This shift in power balance between the audience and speakers enables easier interactions and the exchange of ideas in discussion, augmenting caregivers’ understanding and interest in health-related topics.

From this, SC has empowered the audience to voice their questions and concerns, and to use their knowledge to improve not only to their own family’s health but also to spread this knowledge to the community they live in. As explained by this participant:

Very helpful because we know more about how to prevent illness in our children and can share this information with the other families in our village.

(Science café, Audience)

The YPAG programme empowered its members through encouraging them to be more confident and responsible. The questionnaires showed that the majority of YPAG members and their parents thought that the young people had gained these attributes after participation in YPAG. YPAG members expressed that they felt more empowered:

In YPAG I have had opportunities to decide what I think is important to do (with regard to YPAG activities) by myself, and then do it.

(YPAG member)

Changing attitudes towards sharing knowledge and expressing one’s views is an essential step in empowering community members so that they are able to take an active role in shaping the care provided by the hospital and in health practices within their community. Such changes in practice are only possible if there is an established degree of trust in the organisation delivering the engagement programme, as discussed in the section below.

### Trust

Participants referred to trust of AHC (organisational trust) and of the organiser (individual trust) which will be discussed in turn.

### Organisational trust

One manner in which the hospital community engagement programme has gained trust from community members is through teaching and raising awareness. SC audience members were clear that they appreciated the teaching delivered:

I like it very much because I can gain new knowledge from doctors and nurses.

(Science Café, Audience)

As shown from the questionnaire results, almost all YPAG members and their parents said that they trust AHC (Fig 1).

When asked why they want their children to be part of the YPAG the responses given by the YPAG parents demonstrate there is a degree of trust towards AHC and the organisation of YPAG. They said they believe YPAG provides a good environment for their children.

I don't want them to hang out with bad friends, that’s why I want my child to join YPAG.

(Parent of YPAG member)

The impact of the teaching provided by the hospital community engagement programme was clear from participants’ responses. This quote from an SC audience member illustrates that the SC has been successful in imparting knowledge and that that increased knowledge has an impact on healthcare practices:

The things I learnt will help me in making better decisions and in giving the correct medicine to my child.

(Science café, Audience)

Responses, such as in the quote below, from YPAG members also indicated the benefit of the programme in imparting knowledge and increasing their confidence.

Because YPAG helps boost my confidence and improves my knowledge.

(YPAG member)

### Individual trust

Further to establishing organisational trust, for the YPAG it was essential to also establish individual trust of the organiser with the participants. The organiser plays a key role in the YPAG programme, with intensive contact with the participants, as a facilitator and supervisor. As such, the success of the programme relied upon the young people and their parents having individual trust in the organiser.

My parents and the YPAG facilitator are the reasons that I come here.

(YPAG member)

Because I want my child to be more responsible and share their knowledge and views with the team. I thank YPAG for giving my child the chance to make this happen.

(YPAG, Parent)

Trust is key for participants to engage in the programme in such a way that they derive benefit from it. Increasing knowledge and confidence are examples of such benefits, and demonstrate that participants value the hospital community engagement programme.

### Communication

The hospital community engagement programme hinges on good communication, which is vital in narrowing the gap between the community members and the hospital. Throughout the design and implementation of the YPAG and SC effective communication was given priority, with a core value of the programmes being to identify and address the needs of the community, delivering information in a way that meets their needs.

For this reason, the SC was implemented in a relaxed environment that allowed everyone to communicate easily with each other. Another factor in achieving this is that the presentations were given in Khmer and the slides contained only pictures. This audience member explained how this made them feel:

It was very easy to understand because the presenter spoke slowly enough that I could understand. Even though the topic was hard and scary the way of explaining made it understandable.

(Science Café, Audience)

Often, audience members said that the way in which presenters communicated was easy to understand. The relaxed and positive demeanour of the presenters and their ability to communicate scientific concepts in lay terms allowed audience members to understand the topic well and quickly. This meant that, during the Science Café sessions, there were a lot of discussions among the audience and presenters, augmenting their understanding of the topic.

This manner of communication allowed all audience members to feel included, regardless of their level of education and literacy. Every audience member could listen and look at the slides without feeling undermined. This in turn increased the audience members’ trust in SC, the organisation and presenters, such that they were more receptive to the messages delivered.

I found it very helpful because when they explain the topic well it makes me want to listen so that I could learn more. It was well explained and the presenter was very polite and very nice.

(Science Café, Audience)

Good communication is a key feature of the YPAG also, in terms of the organiser communicating with and bringing together a group of 20 young people the majority of whom did not know each other previously, in such a way that they are now able to work very effectively together as a team, and indeed are friends. Both the YPAG members and their parents recognise good communication skills, friendship and unity of the YPAG as essential benefits imparted by participation in the programme:

I want YPAG to continue for a long time and I want our team to continue to be strong together so our friendship can last longer.

(YPAG member)

I believe in AHC as my child is now able to express himself better and is more responsible.

(YPAG, Parent)

The Executive Committee also recognised the importance of effective communication with regard to caregivers and children, and also with regard to hospital staff involved in the programme, providing lasting benefit to AHC:

This is also a chance for the junior doctors and nurses to improve their communication skills.

The science café will soon reduce the gap between the hospital’s needs and the public’s needs.

(Science Café, Executive Committee members)

Developing as a team also requires good communication skills amongst the YPAG members themselves: to communicate their views and negotiate disparate views within a group to achieve a common goal. This will be explored in the section below.

### Teamwork

One of the objectives of the YPAG was to build the capacity of the young people to work together as a team. The YPAG members revealed that they have changed a lot through working as a team including being able to share their views, gaining more friends and having more experience in conducting activities as a team.

I don't feel lonely anymore because now I have more friends from YPAG.

(YPAG, Children)

He has changed from a child who did not speak much to becoming more confident and reasoned….and he now understands what volunteer work is.

(YPAG, Parent)

The activities undertaken by the YPAG were suggested by the organiser and agreed upon by the group. Their intended benefit was to enhance team-working skills and engagement in AHC, and to be of importance to AHC. One unintended consequence is that in sharing the aspect of being of benefit to others, the YPAG activities developed the concepts of social responsibility and altruism, as mentioned in the above quote. Other unintended consequences will be discussed in the section below.

### Unintended consequences

The hospital community engagement programme had certain goals it wished to achieve with community members, such as increasing knowledge and involvement in AHC. Further benefits were revealed when participants were asked if they have seen changes in themselves. The YPAG members feel more confident, more responsible and think that their voice is being heard. Most of their parents mentioned that their children are now more confident than before in terms of sharing opinions, studying harder, and are more organised.

An important unintended consequence emergent throughout the hospital community engagement programme is that all participants reported developing greater responsibility. Through SC, caregivers gained greater knowledge and were empowered to take responsibility for the care of their children, for example by bringing them to hospital appropriately.

I know more about how to detect pneumonia and I know when I have to bring my children to the hospital, and make sure I don’t delay in bringing them.

(Science café, Audience)

During YPAG meetings participants take turns in leading the team, with different groups responsible for certain tasks such as arranging the meeting, setting up the room and taking attendance. The groups would rotate so that everyone would share the different responsibilities and have the chance to be the team leader. In doing so, the group were able to work together, develop leadership skills, and take responsibility for the group. Furthermore, in conducting activities to benefit others (such as the patient satisfaction survey), the YPAG members were taking responsibility to improve the quality of healthcare delivered to their community’s children. Both the YPAG members and their parents spoke of the importance of developing this greater sense of responsibility amongst the young people.

Through YPAG I want my child to learn to recognise their mistakes and to take responsibility for their actions.

(YPAG, Parent)

Another unintended benefit was that in addition to being useful, participants agreed that the hospital community engagement programme was enjoyable. All of the participants involved in the SC and YPAG said they liked the events because they are fun and relaxed. The presenters also found the events enjoyable, in particular the interaction with the audience. Providing food created a more relaxed environment, and was appreciated by all.

These unintended benefits of developing confidence, responsibility and enjoying participation help the community engagement programme to empower participants and result in lasting change in community healthcare practices. Respondents also gave suggestions for how the programme could be improved, discussed in the section below.

### Suggestions for improvement

There were different suggestions for improvement from the different stakeholders regarding the hospital community engagement programme’s activities. Science Café audience members requested more frequent meetings so that more people could learn and enjoy the events. YPAG members and their parents wanted to build on the attributes the group is developing, to be able to express themselves better, to be more confident and more responsible, and they want the YPAG to be better known within the wider community.

I really want my child to be more independent and to be better able to communicate within a group.

(YPAG, Parent)

Suggestions from the AHC Executive Committee included ensuring that YPAG members include people form a variety of backgrounds, such that a range of views from Cambodian children can be gathered.

Make sure the group is "representative" of Khmer society.

(YPAG, Executive Committee member)

Executive Committee members valued the input from YPAG and wanted frequent regular surveys:

To do regular surveys so that they can get clear recommendations from the children's perspective on hospital services and infrastructure and ways in which we can make their stay as good as possible.

(YPAG, Executive Committee member)

It can be seen from the above suggestions that all key stakeholders value the hospital community engagement programme, wishing to build on it further.

## Discussion

The aims of the hospital community engagement programme of empowering community members to be familiar with science topics and actively involved in AHC, in their own and their children’s health are being met as reported by participants in this evaluation. The two component programmes, SC and YPAG, involved adults (caregivers of admitted children) and young people respectively to holistically engage the recipient stakeholders within AHC, with common programme goals of participant empowerment, awareness raising, good communication and building trust within a team and with AHC. Science Café has empowered caretakers to be able to appropriately recognise signs of illness in their children and to improve their own health through effective communication in a manner suited to their needs, and creating an environment in which they can confidently ask questions to enhance their understanding. The YPAG has empowered the young people to communicate their views, build friendships and work effectively as a team so that they can participate in discussions, allowing them to gain a wider view of important healthcare issues for the community and be actively involved in the healthcare decision-making within AHC.

Limitations of this study include that it involved responses from participants only, and therefore is unable to determine the views of the wider community. Additionally, participants are self-selected and may not be fully representative of the community. In a similar vein, this early-stage evaluation can only conclude the short-medium term outcomes of the hospital community engagement programme. A longer period of time is required for the programme to impact on the wider community, which is something that can be addressed during another evaluation at a later stage. The current evaluation is essential in the iterative development of the programme and shows that it is currently meeting its goals.

The hospital community engagement programme is unique to this setting, and is proving successful for a variety of reasons. The programme was created to meet the needs of its target audience, and as such was designed to be non-hierarchical and relaxed. The importance of delivering an enjoyable and sociable programme was recognised, to help participants feel relaxed and able to engage effectively. One of the ways this was achieved was by providing food, to create a relaxed environment for discussions.

It was essential to develop an environment of non-hierarchical interactions. Participants recognised this deliberate shift to equalising the power balance during the events, and felt able to voice their opinions and ask questions back and forth comfortably. Moreover, effective communication allowed all participants to be involved. For SC this was achieved by using slides with only pictures and delivering the presentation in Khmer so that participants from any educational background could understand without feeling undermined. For the YPAG, the activities were implemented to facilitate an equal sharing of views amongst participants, and collaboration through teamwork.

A vital factor in the ability of the hospital community engagement programme to impact on its participants is that there was trust in the organisation and in the organiser [13]. This trust was fundamental in achieving a relaxed environment, and in participants wanting to be involved, without which the programme could not have had the impact it has.

The importance of the hospital community engagement programme and its ability to impact on improving healthcare in the community was realised by all stakeholders, which is key in its success so far. The commitment from the AHC Executive Committee facilitated the smooth running of the programme, with Executive Committee members believing the programme to be an effective way of bridging the gap between the community members and the hospital. Similarly the commitment of participants has enabled the programmes to be useful and successful in imparting knowledge and developing essential attributes to empower community members.

One example of how these factors together result in an empowered group is that of the garden playground. The YPAG decided to conduct a patient satisfaction survey, during which they found that the majority of patients wanted a new playground. This was part of their presentation to the Executive Committee. As a result, the committee decided to introduce a new playground. The YPAG have been critically involved through the design stages, for example in that the playground must still include the garden area, which is valued by patients. As such, the garden playground will be a direct result of the YPAG’s voice being heard by the Executive Committee, resulting in a better experience for patients.

## Conclusion

A hospital community engagement programme must be delivered in the right way to meet the needs of community members, including right communication, right setting, right people and right timing. Key aspects of a hospital community engagement programme include effective communication and open channels of discussion so that community members feel comfortable to ask questions. Equally important are developing trust between community members and the organisation and the individuals delivering the programme, by delivering it in a comfortable space and in a non-hierarchical manner. Essential attributes that such a programme can develop in participants are teamwork and passion, such that they are able to work together to improve their own health. These attributes, combined with greater knowledge about health-related topics will allow community members to feel empowered and confident to disseminate this knowledge within their community. Underpinning all of this is a high level of commitment to the programme by the senior management of the hospital. In these ways the gap between hospitals and the community could get smaller, allowing everyone to interact and learn from each other.

In order to have a successful hospital community engagement programme, understanding the target audience is essential. Putting the needs of the community members first allows engagement programmes to be designed and delivered in a way that is appropriate and effective for them. This ultimately results in a meaningful programme that is able to empower community members, potentially resulting in lasting change in healthcare practices.
